# Direct Coronary Vascular and Lipidomic Remodeling by Low-Dose GLP-1 Agonist Therapy in Cardiometabolic HFpEF

**DOI:** 10.21203/rs.3.rs-10630296/v1

**Published:** 2026-08-11

**Authors:** David Lefer, David Polhemus, Mahmoud Elbatreek, Craig Resch, Annie Butt, Faisal Rahman, Cinthia Drachenberg, David Burk, Timothy Allerton, Amir Ravandi, Traci Goodchild

**Affiliations:** Cedars-Sinai Medical Center; Johns Hopkins University School of Medicine; Cedars-Sinai Medical Center; University of Manitoba; Johns Hopkins University School of Medicine; Johns Hopkins University School of Medicine; University of Maryland; Pennington Biomedical Research Center; Pennington Biomedical Research Center; University of Manitoba; Smidt Heart Institute

## Abstract

Glucagon-like peptide-1 receptor agonists (GLP-1RAs) reduce major adverse cardiovascular events in patients with type 2 diabetes and obesity[1, 2], but the mechanisms underlying these benefits remain incompletely defined. Although GLP-1RAs improve vascular function[3] and alter circulating lipid species associated with cardiovascular risk [4–6], whether these effects extend to diseased coronary vessels and occur independently of weight loss is unknown. Here, we show that weight-neutral semaglutide administration in two complementary cardiometabolic HFpEF models - a porcine model of metabolic syndrome with coronary artery disease and the ZSF-1 obese HFpEF rat - improves coronary and aortic endothelium-dependent vasodilation, attenuates coronary vascular remodeling and fibrosis, increases myocardial microvascular density, and remodels the plasma lipidome through reductions in ceramides and diacylglycerols together with enrichment of long-chain polyunsaturated triacylglycerols. These findings identify direct vascular and metabolic actions of semaglutide that are independent of weight loss.

## Introduction

Glucagon-like peptide-1 receptor agonists (GLP-1RAs) have emerged as transformative therapies for cardiovascular risk reduction. Landmark clinical trials have demonstrated that semaglutide significantly reduces major adverse cardiovascular events (MACE) in patients with type 2 diabetes and in individuals with obesity without diabetes [1, 2]. While weight loss and glycemic control contribute to these benefits, accumulating evidence suggests that GLP-1RAs exert vascular effects that may be independent of these systemic metabolic improvements. Despite these clinical benefits, major questions remain in our understanding of GLP-1RA cardiovascular pharmacology. To our knowledge, no study has comprehensively evaluated the effects of GLP-1RAs on coronary endothelial function and coronary remodeling in established coronary artery disease. Whether these beneficial vascular or lipidome remodeling effects occur independently of weight loss remains unclear because weight-neutral dosing has rarely been explored. The present study addresses these gaps by employing two complementary preclinical models: a porcine model of metabolic syndrome and HFpEF with coronary artery disease and the ZSF-1 obese rat model of cardiometabolic disease. We administered low-dose semaglutide and performed comprehensive assessments of coronary vascular histopathology, vascular function, and plasma lipidomic profiles (>1,200 lipid species across 17 lipid classes) to define vascular and metabolic effects of GLP-1RA therapy independent of weight loss.

## Results

### Weight-neutral semaglutide does not alter body weight in two cardiometabolic HFpEF models.

Female Göttingen minipigs underwent a “two-hit” HFpEF induction protocol consisting of deoxycorticosterone acetate (DOCA) administration and an 8-week Western diet to generate metabolic syndrome with coronary artery disease (Figure 1a). Animals were then randomized to semaglutide (titrated to a maintenance dose of 35 μg/kg, intramuscular, weekly) or vehicle for 12 weeks. Semaglutide did not significantly alter body weight throughout treatment (43.5 ± 2.7 kg in the vehicle group and 46.6 ± 2.9 kg in the treatment group, adjusted p>0.99; at the study endpoint). Coronary angiography showed luminal stenosis characteristic of this model (Figure 1b). At study completion, semaglutide-treated pigs had markedly reduced epicardial adipose tissue surrounding the coronary arteries compared with vehicle-treated animals (Figure 1c).

### Semaglutide improves endothelial function across species.

In isolated porcine LAD and left circumflex (LCX) coronary artery rings, semaglutide significantly enhanced endothelium-dependent relaxation to bradykinin (BK) and substance P (SubP), compared with vehicle (Figure 1d). In contrast, endothelium-independent relaxation to sodium nitroprusside (SNP) was unchanged. Thoracic aortic rings from ZSF-1 obese rats treated with semaglutide (125 μg/kg twice weekly) or vehicle for 16 weeks were also examined. Body weight did not differ between groups throughout the study (612.5 ± 12.28 g in the vehicle group and 558.9 ± 12.39 g in the treatment group, adjusted p = 0.09 at the study endpoint). In the rat aorta, semaglutide similarly enhanced acetylcholine (Ach)-mediated relaxation without altering responses to SNP (Figure 1e).

### Semaglutide attenuates coronary ultrastructural pathology, medial remodeling, and fibrosis.

Coronary arteries from vehicle-treated pigs exhibited extensive ultrastructural abnormalities on transmission electron microscopy, including intracellular lipid vacuoles, lipid-laden macrophages, interstitial edema, and cellular debris (Figure 2a). Histologic analysis showed advanced coronary disease characterized by intimal hyperplasia and luminal narrowing (***Extended Figure 1a-h and Extended Table 1***). Quantitative morphometry and blinded histologic scoring demonstrated that semaglutide numerically reduced intimal volume compared with vehicle (Figure 2b,e,f; ***Extended Table 1***). picrosirius red staining showed significantly less perivascular fibrosis in semaglutide-treated coronary arteries (Figure 2c,g), whereas coronary calcification was numerically lower but did not reach statistical significance (p=0.07) (Figure 2d,h). Capillary density was quantified by CD31 immunostaining. Semaglutide-treated pigs exhibited greater CD31-positive microvascular density and red blood cell count than controls (***Extended Figure 2a-d***).

### Semaglutide induces broad lipidome remodeling in ZSF-1 obese rats.

Untargeted plasma lipidomics quantified 1,203 lipid species across 17 lipid classes. Semaglutide induced extensive remodeling of the plasma lipidome, with 445 of 1,203 species (37.0%) differing between groups after false discovery rate (FDR) correction (q < 0.05) (Figure 3a). Among significant species, 187 were increased and 258 were decreased, with log_2_ fold changes ranging from −2.80 to +6.28. Triacylglycerols (TAGs) exhibited the greatest degree of remodeling, with 276 species reaching FDR significance (median log_2_FC = +0.72) (Figure 3b). In contrast, phosphatidylglycerols (PGs) and phosphatidylcholines (PCs) were predominately reduced, with all 26 significant PG species and 17 of 17 significant PC species decreased. Cholesteryl esters (CE) were the only other lipid class showing predominantly increased abundance, with all 5 significant species elevated. All 13 FDR-significant ceramide species were reduced (median log_2_FC = −0.88), including CER(d18:0/18:0) and CER(d18:1/16:0) (Figure 3c). Similarly, all 11 significant sphingomyelin species and all 69 significant diacylglycerol (DAG) species were decreased (median log_2_FC = −0.82 and −0.67, respectively) (Figure 3c–d). Within the TAG class, the largest increases occurred in polyunsaturated species containing docosahexaenoic acid (DHA), including TAG(54:4/FA20:4), TAG(56:7/FA22:6), and TAG(54:4/FA22:4). Median TAG abundance increased progressively with acyl chain unsaturation, from +0.16 for saturated TAGs to +1.59 species containing ≥5 double bonds. TAG treatment effects were positively correlated with both total acyl carbon number (r = 0.54, p < 0.001) and total double bond number (r = 0.35, p < 0.001) (Figure 3e–f).

### Semaglutide produces a concordant lipidomic signature in the porcine HFpEF model.

Development of the porcine cardiometabolic disease model induced widespread plasma lipid accumulation, while cholesteryl esters and sphingolipids (including ceramides and sphingomyelins) were among the most prominently increased lipid classes (***Extended Figure 3a-b***). Circulating total cholesterol was also markedly elevated by 8 weeks and remained similarly elevated by the study endpoint (***Extended Figure 3c***). To evaluate semaglutide-induced lipid remodeling, post-treatment changes were calculated relative to the pre-treatment (8-week timepoint) for each animal. Owing to the smaller sample size (n=5 per group), no lipid species remained significant after FDR correction; however, 76 species reached nominal significance (p<0.05). Despite the limited sample size, similar directional changes were observed in ZSF-1 rats (Figure 4a–b). All 12 nominally significant TAG species were increased, with the largest effects occurring in DHA-enriched polyunsaturated TAGs. Across all 445 TAG species, treatment effects increased with both acyl chain length and degree of unsaturation, consistent with the pattern observed in rats (Figure 4c–d). Semaglutide also reduced glucosylceramides (GlcCer), with all 5 nominally significant GlcCer species decreased, 14 of 19 ceramide species showed directional decreases despite not reaching individual statistical significance. In addition, DHA-containing PCs and PGs were preferentially increased. Unlike the ZSF-1 obese HFpEF rat model, DAGs exhibited minimal overall change, with only 2 species reaching nominal significance.

## Discussion

In two complementary preclinical models of cardiometabolic HFpEF, we demonstrate that semaglutide exerts direct cardiovascular effects independent of weight loss. Weight-neutral semaglutide improved endothelial function, attenuated pathological coronary artery remodeling, reduced coronary perivascular fibrosis, and increased myocardial microvascular density. These vascular changes were accompanied by broad plasma lipidome remodeling characterized by reductions in ceramides and enrichment of polyunsaturated docosahexaenoic acid (DHA)-containing triacylglycerols (TAGs), a lipidomic signature that was conserved across rat and pig HFpEF models. Together, these findings separate vascular and lipidomic effects of semaglutide from its effects on body weight, suggesting that GLP-1 receptor activation modifies cardiovascular biology through mechanisms beyond weight reduction.

### Establishment of a Large Animal Pre-Clinical Model of Coronary Disease in HFpEF

The Göttingen swine DOCA/Western diet model provides a translational platform for studying coronary vascular pharmacology in the setting of severe cardiometabolic disease and HFpEF. We previously reported that this model develops HFpEF with diastolic dysfunction, elevated left ventricular filling pressures, and myocardial fibrosis [7] resulting from hypertension and metabolic syndrome. Here, we show that it also recapitulates key features of human coronary artery disease, including endothelial dysfunction, intimal hyperplasia, luminal narrowing, calcification, and adverse coronary remodeling[8–10]. Given the close anatomical and physiological similarity between porcine and human coronary arteries, this model provides an important bridge between rodent studies and clinical investigation.

### Weight-Neutral Semaglutide Improves Coronary Vascular Reactivity and Remodeling

A central finding of this study is that semaglutide improved endothelium-dependent vasodilation while preserving endothelium-independent vascular responses, indicating selective improvement in endothelial function. These findings were observed in both porcine coronary arteries and rat aortas, suggesting that the vascular effects of semaglutide are reproducible across species and vascular beds. This is consistent with previous studies demonstrating that GLP-1RA enhances nitric oxide bioavailability [11] and non-coronary vascular function[12], and may represent a mechanism through which GLP-1RAs improve vascular health. Improvement in endothelial function was accompanied by less perivascular fibrosis and greater myocardial microvascular density. Although these endpoints measure different aspects of vascular biology, the concordance of functional and structural changes suggests that semaglutide influences multiple components of the coronary vasculature. Clinical trials demonstrate cardiovascular benefit that exceeds what would be expected from glycemic control alone. Our findings identify coronary endothelial function and vascular remodeling as candidate mechanisms that may contribute to those clinical effects.

### Protective Remodeling of the Plasma Lipidome

Plasma lipidomics detected widespread remodeling despite the absence of weight loss. Rather than inducing uniform lipid changes, semaglutide selectively remodeled lipid composition, enriching polyunsaturated DHA-containing TAGs while reducing ceramides, sphingomyelins, and diacylglycerols. The enrichment of polyunsaturated TAG species was independently reproduced in both species despite substantial differences in metabolic background, study design, and statistical power, suggesting that this represents a conserved metabolic response to GLP-1 receptor activation. Earlier lipidomic studies primarily described changes in selected lipid classes after GLP-1RA treatment [6, 13]. In contrast, untargeted profiling identified coordinated remodeling across more than 1,200 lipid species, with particularly consistent enrichment of long-chain polyunsaturated TAGs in both models.

### Ceramide Reduction and Coronary Risk

Among the lipid changes observed, the reduction in ceramides is particularly notable given their established association with endothelial dysfunction, insulin resistance, and adverse cardiovascular outcomes [14]. In rats, all significantly altered ceramide species were decreased, while the porcine model demonstrated reductions in glucosylceramides together with similar directional changes across most ceramide species. These findings extend previous reports of reduced circulating ceramides following liraglutide treatment in patients with type 2 diabetes by demonstrating comparable sphingolipid remodeling under weight-neutral conditions [4, 5]. Whether these lipid changes directly mediate the vascular improvements observed in this study remains unknown; however, the concordance of endothelial, structural, and lipidomic remodeling across two independent models raises the possibility that altered sphingolipid metabolism contributes to the vascular phenotype observed after semaglutide treatment.

### Limitations

Several limitations warrant consideration. First, the porcine study was limited by a modest sample size (n = 5 per group), reducing statistical power to detect smaller treatment effects, particularly within the lipidomics analyses. Second, plasma lipidomics were performed at a single post-treatment timepoint, precluding assessment of the temporal relationship between lipid remodeling and vascular improvement. Third, vascular function was assessed *ex vivo* and therefore does not fully capture the influence of *in vivo* hemodynamics or neurohumoral regulation. Finally, this study does not establish a causal relationship between lipid remodeling and improved vascular function. Future mechanistic studies will be required to determine whether alterations in sphingolipid metabolism directly contribute to the vascular protection conferred by GLP-1 receptor agonists.

## Methods

### Porcine model of metabolic syndrome and coronary artery disease.

Female Göttingen minipigs (12 months old) were obtained from Marshall Bio-Resources (Rose, NY). The animals underwent a “two-hit” cardiometabolic disease and HFpEF induction protocol based on previously published methods with minor modifications[7, 15]. To induce hypertension, 10 minipigs received subcutaneous deoxycorticosterone acetate (DOCA) pellets (75 mg/kg 90-day 200 mg release pellets; Innovative Research of America, Sarasota, FL). Concurrently, obesity and a pre-diabetic state were induced by feeding the animals a custom apple flavor Western diet (5ZVP TestDiet; 4.04 kcal/g) consisting of 37.1% carbohydrates (including 9% high-fructose corn syrup), 18.1% protein, 44.8% fat, 1% cholesterol, 0.7% sodium cholate, and 2% total salt. Water was provided *ad libitum*. After 8 weeks of disease induction—at which point the minipigs were 14 months old and displayed echocardiographic evidence of diastolic dysfunction (increased E/e’ ratio)—the animals were randomized into two groups (n = 5 per group) for a 12-week treatment period: Control: Received no specific treatment, and Semaglutide: Received weekly intramuscular injections of semaglutide (Cat No. 40170, Cayman Chemical), up-titrated over a 4-week period from 2.2 μg/kg to 17.5 μg/kg, followed by a maintenance dose of 35 μg/kg for the remaining 8 weeks. A companion manuscript using the same experimental cohort reports the effects of low-dose semaglutide on HFpEF and cardiac and hepatic metabolism; the present study presents distinct analyses focused on coronary vascular and circulating lipid remodeling.

Investigators performing functional and histological assessments were blinded to treatment allocation. Body weight was assessed weekly. At endpoint, animals were anesthetized, coronary angiography was performed, and hearts were excised for *ex vivo* vascular reactivity, histology, and TEM. Blood was collected at endpoint into EDTA-coated tubes; plasma was separated by centrifugation and stored at −80°C until lipidomic analysis.

### ZSF-1 obese rat model.

ZSF-1 obese rats, a genetic model of obesity, insulin resistance, hypertension, and heart failure with preserved ejection fraction, were used. Male ZSF-1 obese rats (10 weeks old at baseline) were randomized to receive either Vehicle (PBS with 0.05% Tween 80) or semaglutide (125 μg/kg, subcutaneous, twice weekly) for 16 weeks (n = 6 per group). This dose was empirically determined in a pilot study to be a non-anorectic dose that provides systemic GLP-1 receptor engagement without inducing significant weight loss in this hyperphagic strain. Body weight and metabolic parameters were assessed weekly. At endpoint, rats were anesthetized, thoracic aortas were excised for ex vivo vascular reactivity, and blood was collected into EDTA-coated tubes; plasma was separated and stored at −80°C until lipidomic analysis. All animal procedures were approved by the Institutional Animal Care and Use Committee (IACUC) of Cedars-Sinai Medical. As with the porcine model, a companion manuscript using the same experimental cohort reports the cardioprotective and hepatoprotective effects of low-dose semaglutide. The present study presents distinct analyses. Aortic vascular reactivity data were presented in the companion manuscript in a separate cohort of ZSF-1 obese rats.

### Coronary angiography.

Coronary angiography was performed in anesthetized minipigs at the study endpoint. Fluoroscopic imaging was used to visualize the coronary tree following intracoronary contrast injection. Coronary analysis was performed by a board-certified interventional cardiologist (FR) blinded to treatment group assignment.

### Ex vivo vascular reactivity.

Coronary artery segments (LAD and LCX from pigs) and thoracic aortic rings (from rats) were dissected free of adherent tissue and mounted in an organ bath system containing oxygenated Krebs-Henseleit buffer at 37°C. After equilibration at appropriate passive tension, vessels were contracted with phenylephrine to achieve approximately 80% of maximal contraction. Cumulative concentration-response curves were constructed for bradykinin (10^−10^ to 10^−6^ M), substance P (10^−10^ to 10^−6^ M), acetylcholine (10^−9^ to 10^−5^ M), and SNP (10^−10^ to 10^−5^ M). Responses were expressed as percentage relaxation of the precontraction state. Vascular reactivity experiments were performed by an investigator blinded to treatment group.

### Histology and histomorphometry.

Coronary artery segments and left ventricle tissue were fixed in 10% neutral buffered formalin, paraffin-embedded, and sectioned at 5 μm. Sections were stained with hematoxylin and eosin for morphometry and picrosirius red for collagen. Medial thickness, lumen diameter, and media-to-lumen ratio were measured in H&E-stained LAD cross-sections using automated image analysis (QuPath/ImageJ). Perivascular fibrosis was quantified as percentage collagen-positive area in Sirius red-stained sections using automated image analysis (QuPath/ImageJ). Analysis was performed by an investigator blinded to treatment group. Histology scoring of H&E stained coronary artery sections was performed in a blinded fashion by a board-certified clinical cardiac pathologist (CD). For CD31+ staining, slides were baked for 30 min @ 60C prior to staining. After transferring to the Bond, the slides were dewaxed, subjected to heat-induced antigen retrieval for 40 min with Leica ER2 buffer, and incubated with anti-CD31 (Abcam ab182981, 1:1000) for 60 min @ 40C. After washing, the slides were then incubated with Vector Horse anti-Rabbit HRP polymer (Vector, MP-7401) and streptavidin-Cy5 (Jackson Immunoresearch, 016–170-084) for 30 min at room temperature and finally incubated with CF594-tyramide (Biotium,92174) for 10 min before counterstaining with Hoechst.

### Transmission electron microscopy.

LAD coronary artery segments were fixed in 3.0% glutaraldehyde in 0.1 M sodium phosphate buffer (pH 7.3), post-fixed in 1% osmium tetroxide, dehydrated through graded ethanols, and embedded in Epon resin (PolySci). Samples were polymerized overnight at 60°C. Ultrathin sections (60–90 nm) were cut with a diamond knife on a Leica EM UCT ultramicrotome and collected on 2×1 mm Formvar-coated copper slot grids. Grids were stained with 3% uranyl acetate followed by lead citrate and imaged on a ThermoFisher Talos L120C transmission electron microscope at 120 kV using a ThermoFisher Ceta 16 MP CMOS camera. For each sample, five random fields were imaged at 2,000× and 11,000× magnification by an investigator blinded to treatment group.

### Mass spectrometry-based lipidomics.

Plasma lipids were extracted using a modified Folch method. Briefly, 10 μL of plasma was added to 1 mL chloroform/methanol (2:1, v/v), followed by 90 μL PBS and 10 μL of a 1:10 dilution of UltimateSPLASH ONE internal standards (Avanti Polar Lipids) at a final concentration of 0.3 nmol/mL. Samples were vortexed and centrifuged to induce phase separation; the organic layer was collected, dried under nitrogen, and reconstituted in 50:50 (v/v) butanol/methanol. Extracts were stored at −80°C until analysis. Reverse-phase LC was performed with a Zorbax RRHD Eclipse Plus C18 column (2.1 × 50 mm, 1.8 μm; Agilent) using a UFLC system (Shimadzu) at 300 μL/min with solvent A (tetrahydrofuran/methanol/water, 20:20:60, v/v/v) and solvent B (tetrahydrofuran/methanol/water, 75:20:5, v/v/v), each containing 10 mM ammonium formate. The LC system was coupled to a Sciex 7500+ triple quadrupole mass spectrometer operated in scheduled multiple reaction monitoring (sMRM) mode, targeting 1,203 analytes spanning 17 lipid classes including TAGs, DAGs, ceramides, glucosylceramides, lactosylceramides, sphingomyelins, phosphatidylcholines, phosphatidylethanolamines, phosphatidylglycerols, phosphatidylserines, phosphatidylinositols, phosphatidic acids, lysophospholipids, cholesterol esters, and acylcarnitines. Cardiolipins and free fatty acids were analyzed on a 4000 QTRAP triple quadrupole mass spectrometer (AB Sciex) in sMRM negative ion mode. Lipid abundances were normalized to class-matched internal standards. Quality control filters excluded species with coefficient of variation > 30% in pooled quality control samples or detected in fewer than 80% of samples within any group.

### Lipidomic study design.

Two independent preclinical studies were conducted to evaluate the effects of semaglutide treatment on plasma lipid profiles. The rat study employed an unpaired design comparing semaglutide-treated ZSF-1 obese rats (n=6) to vehicle-treated controls (n=6) at a single timepoint. The pig study utilized a paired design with pre-treatment and post-treatment plasma samples from control (n=5) and semaglutide-treated (n=5) animals. Both studies quantified 1,203 lipid species spanning 17 lipid classes: triacylglycerols (TAG), diacylglycerols (DAG), ceramides (CER), sphingomyelins (SM), phosphatidylcholines (PC), phosphatidylethanolamines (PE), phosphatidylglycerols (PG), phosphatidylserines (PS), phosphatidylinositols (PI), cholesteryl esters (CE), acylcarnitines (ACar), lysophosphatidylcholines (LPC), lysophosphatidylethanolamines (LPE), lysophosphatidylglycerols (LPG), phosphatidic acids (PA), glucosylceramides (GlcCer), and lactosylceramides (LacCer).

### Lipidomic Data Processing and statistical analysis.

All lipid abundance values were log_**2**_-transformed to stabilize variance and reduce distributional skewness before statistical analysis. Raw data were processed in Python using pandas and NumPy. Zero intensity values were replaced with a small constant (1e-6) before log_2_ transformation. For the rat study, between-group comparisons were performed using Welch's two-sample t-test on log_**2**_-transformed values, comparing semaglutide-treated animals to controls. For the pig HFpEF study, a within-animal delta approach was employed to account for inter-animal variability. For each animal and lipid species, the log_**2**_ fold change from baseline to post-treatment was calculated as log_**2**_(post-treatment/pre-treatment). These within-animal deltas were then compared between treatment groups using Welch's two- sample t-test, testing whether the magnitude and direction of change from baseline differed significantly between semaglutide-treated and control animals. Benjamini-Hochberg false discovery rate (FDR) correction was applied in the rat lipidomic dataset. Given the exploratory nature and limited sample size of the pig lipidomic analysis, treatment associated differences are reported using nominal p-values. Adjusted p-values (q-values) were calculated for each lipid species, with q < 0.05 considered statistically significant. Effect sizes were reported as log_**2**_ fold changes. For the rat study, log_**2**_ fold change was calculated as the difference between mean log_**2**_-transformed values in semaglutide-treated and control groups. For the pig study, effect size was quantified as the difference in mean log_**2**_ deltas between treatment groups (mean semaglutide delta minus mean control delta). Class-level summaries report median log_**2**_ fold changes across all species within each lipid class. To evaluate the relationship between lipid acyl chain structure and treatment response, lipid species were categorized by total acyl chain carbon number and degree of unsaturation. Pearson correlation coefficients were calculated to assess associations between total acyl carbon number or total double bond number and log_**2**_ fold change.

To characterize lipidomic remodeling associated with disease progression irrespective of treatment assignment, pig plasma samples from all pigs were pooled and analyzed longitudinally from baseline to week 8. For each lipid species and each animal, the within-animal log_2_ fold-change (log_2_FC) was calculated as log_2_FC = log_2_(week 8 abundance) - log_2_(baseline abundance). To assess whether lipid species had significant changes from baseline to 8 weeks across the cohort, a one-sample t-test was applied to the log_2_FC values for each lipid species, testing the null hypothesis that the mean log_2_FC equals zero. Volcano plots and TAG structural-feature scatter plots were generated with matplotlib. Pearson correlations were computed with scipy.stats. All steps were executed within a single Python workflow.

### Statistics.

Data are presented as mean ± SEM unless otherwise stated. Concentration-response curves were analyzed by two-way ANOVA with Šídák post-hoc correction to compare treatment groups across agonist concentrations. Pairwise comparisons of histomorphometric and gross anatomical endpoints were performed using unpaired two-tailed Student’s t-test. Statistical significance was defined as p < 0.05.

## Supplementary Material

Supplementary Files

This is a list of supplementary files associated with this preprint. Click to download.

- ExtendedTableandFigures.docx

## Figures and Tables

**Figure 1:**
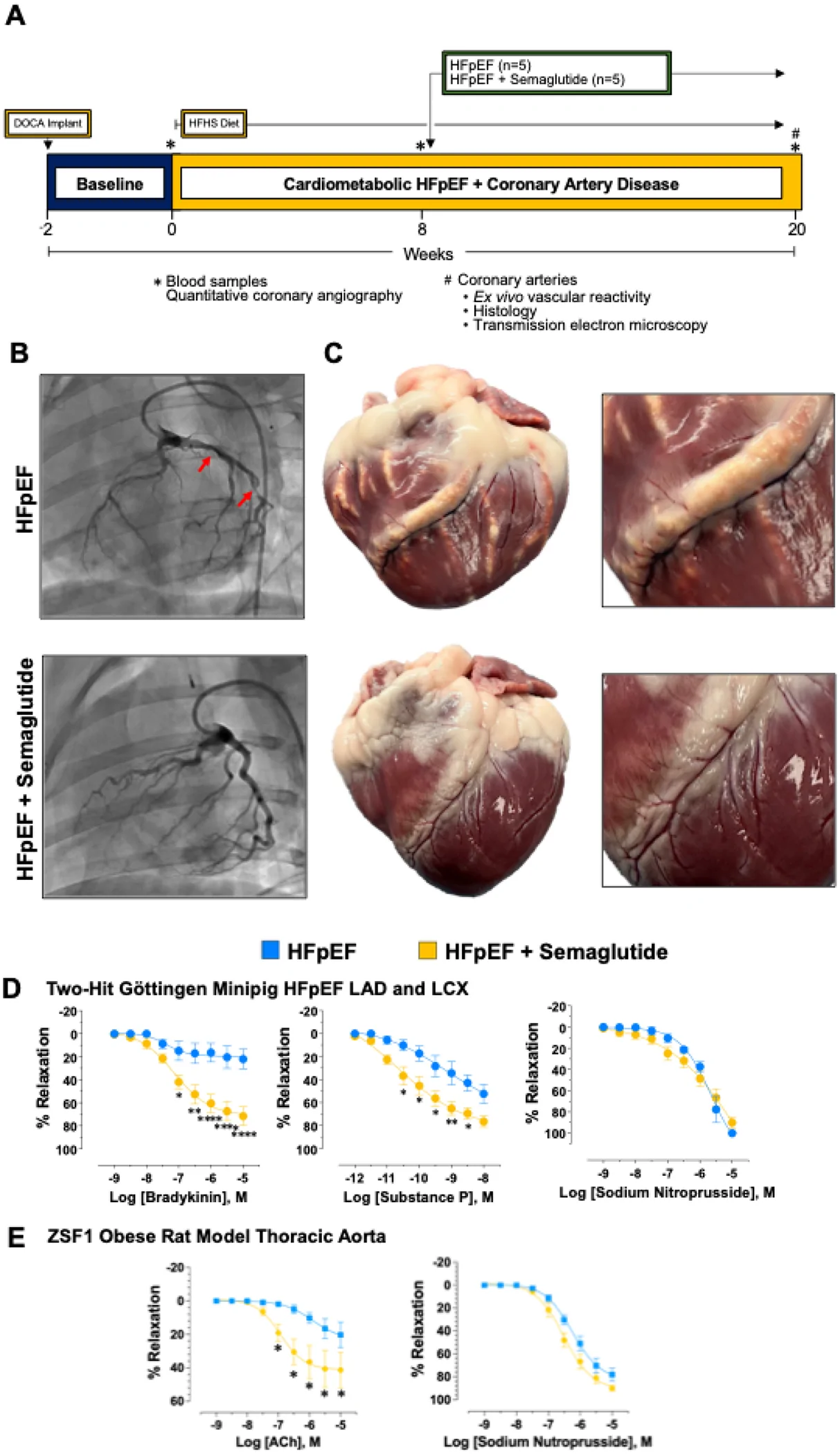
Semaglutide improves coronary and aortic endothelium-dependent vasodilation independent of weight loss. **a**, Experimental protocol schematic for the porcine model of HFpEF and coronary artery disease. Female Göttingen minipigs (n = 5 per group) underwent surgical DOCA pellet implantation and Western diet feeding for 8 weeks (disease induction phase), followed by randomization to vehicle or semaglutide (35 μg/kg intramuscular weekly, titrated to maintenance) for 12 weeks (treatment phase). Endpoint assessments included coronary angiography, invasive hemodynamics, ex vivo vascular reactivity, histology, TEM, and plasma lipidomics. **b**, Representative coronary angiograms at study endpoint. Red arrow denotes significant luminal stenosis in a vehicle-treated control animal. **c**, Representative gross heart photographs at study endpoint. Marked epicardial adipose tissue accumulation overlying the LAD is evident in the control heart (top row); semaglutide-treated heart demonstrates markedly reduced epicardial fat (bottom row). **d**, Ex vivo concentration-response curves for bradykinin (left), Substance P (middle), and SNP (right) in isolated LAD and LCX coronary artery rings from vehicle (blue)- and semaglutide-treated (yellow) pigs (n = 5 animals per group). **e**, Ex vivo concentration-response curves for acetylcholine (left) and SNP (right) in isolated thoracic aortic rings from vehicle- and semaglutide-treated ZSF-1 obese rats (n = 6 animals per group) treated with vehicle (blue) or semaglutide (yellow). Data are mean ± SEM. Two-way ANOVA with Šídák post-hoc correction; *p < 0.05, **p < 0.01 vs. vehicle, ***p < 0.001 vs vehicle, ****p < 0.0001 vs. vehicle at each concentration.

**Figure 2:**
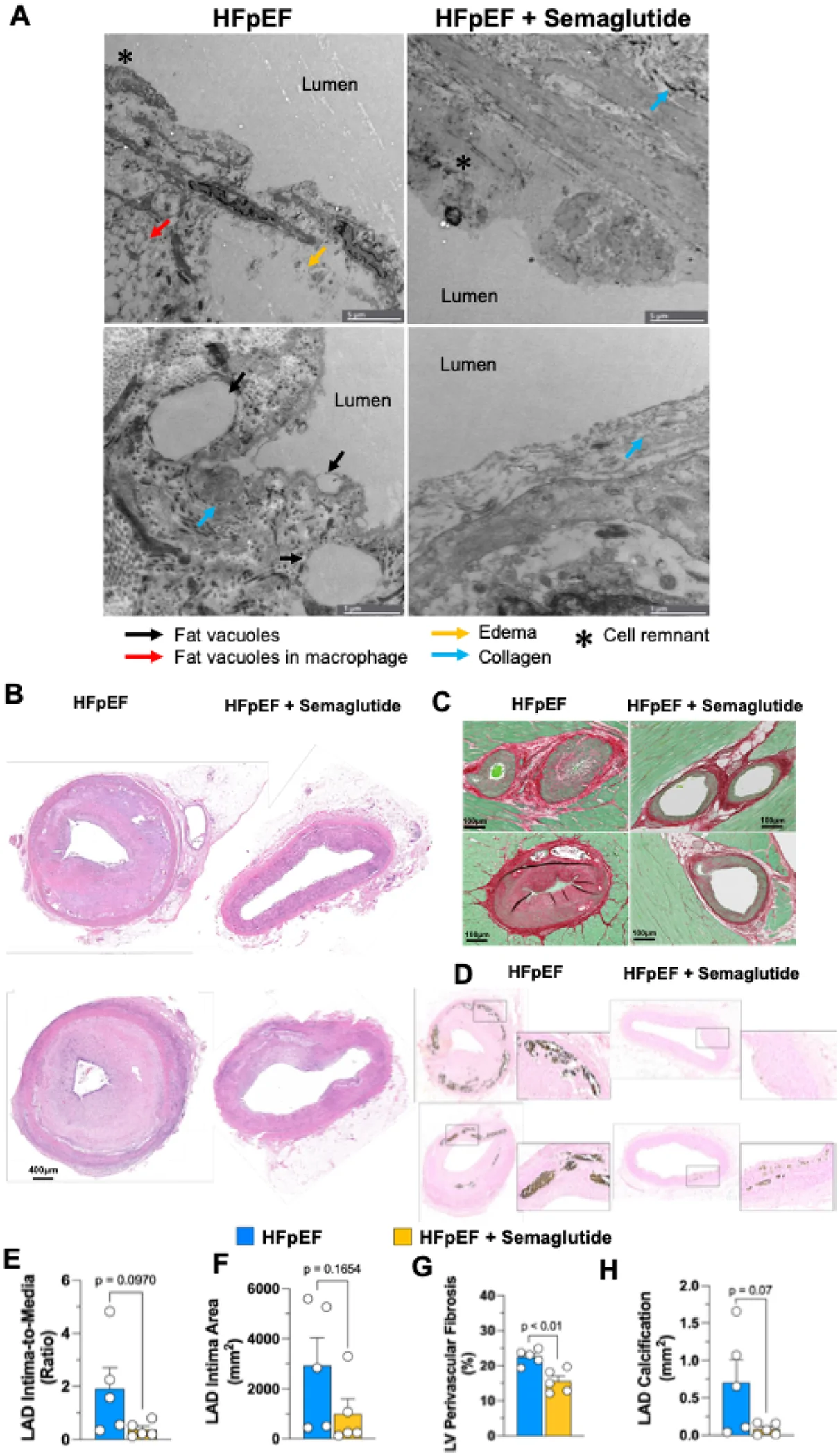
Semaglutide attenuates coronary artery ultrastructural pathology, medial remodeling, and fibrosis. **a**, Representative transmission electron microscopy images of LAD coronary arteries from vehicle- and semaglutide-treated pigs at 2,000× (upper panels) and 11,000× (lower panels) magnification. Intracellular lipid vacuoles (black arrows), lipid-laden macrophages (red arrows), interstitial edema (yellow arrows), collagen deposition (blue arrows), and cellular debris (asterisks) are labeled. Scale bars as indicated. **b**, Representative H&E-stained LAD cross-sections from control HFpEF (left)- and semaglutide (right)-treated pigs. **c**, Representative picrosirius red-stained LAD sections of perivascular fibrosis. **d,** Representative von Kossa-stained LAD cross-sections from control HFpEF (left) and semaglutide (right) treated pigs. **e,** intima-to-media volume ratios and **f,** LAD intima area (mm^2^) calculated from LAD cross sections in HFpEF and HFpEF + Semaglutide treated pigs. **g.** perivascular fibrosis (%) calculated from picrosirius red stain in HFpEF and HFpEF + Semaglutide treated pigs. **h,** LAD calcification volume quantified from von Kossa stained LAD coronary arteries. Data are mean ± SEM; n = 5 animals per group. Unpaired two-tailed Student’s t-test.

**Figure 3:**
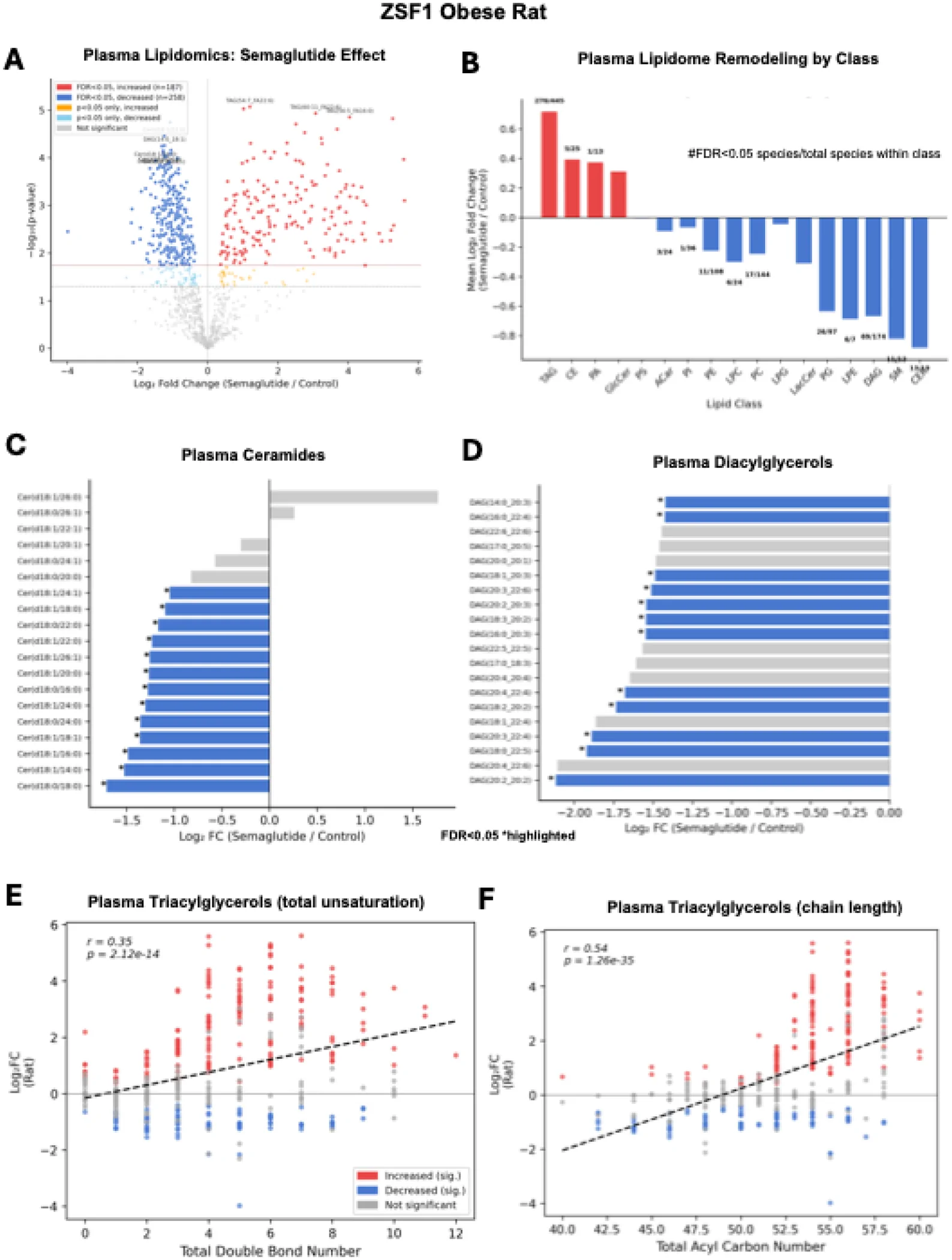
Semaglutide remodels the plasma lipidome in ZSF-1 obese HFpEF rats. **a**, Volcano plot of plasma lipid species abundance comparing semaglutide-treated versus vehicle-treated ZSF-1 obese rats (n = 6 per group). The x-axis shows log_2_ fold change (semaglutide/control) and the y-axis shows −log_10_(p-value). The top horizontal dashed line indicates FDR-adjusted q = 0.05. Data points are color-coded: red dots, FDR < 0.05 and increased in semaglutide (n = 187 species); blue dots, FDR < 0.05 and decreased in semaglutide (n = 258 species); orange dots, p < 0.05 only and increased; green dots, p < 0.05 only and decreased; grey dots, not significant. **b**, Bar chart of mean log_2_ fold change (semaglutide/control) for each of the 17 measured lipid classes. Red bars indicate a net positive mean fold change; blue bars indicate a net negative mean fold change. Numbers above each bar denote the count of FDR-significant species (q < 0.05) relative to the total number of species measured in that class (e.g., 276/445 for TAG). Lipid classes displayed on the x-axis include TAG, CE, PA, GlcCer, PS, ACar, PI, PE, LPC, PC, LPG, LacCer, PG, LPE, DAG, SM, and CER. **c**, Horizontal bar chart of log_2_ fold change for individual ceramide species reaching FDR < 0.05 (n = 13 species). Individual ceramide species are labeled on the y-axis. Blue bars indicate significantly decreased abundance (q<0.05) in semaglutide-treated animals. The x-axis shows log_2_ fold change (semaglutide/control). **d**, Horizontal bar chart of log_2_ fold change for individual diacylglycerol (DAG) species with the greatest log_2_ fold change. Individual DAG species are labeled on the y-axis. Blue bars indicate significantly decreased (q<0.05) abundance in semaglutide-treated animals. The x-axis shows log_2_ fold change (semaglutide/control). **e**, Scatter plot of log_2_ fold change for individual TAG species (y-axis) versus total acyl chain double bond number (x-axis, range 0–12). Data points are color-coded: red, FDR-significant and increased; blue, FDR-significant and decreased; grey, not significant. A linear regression line is overlaid (Pearson r = 0.35, p = 2.12 × 10^−14^). **f**, Scatter plot of log_2_ fold change for individual TAG species (y-axis) versus total acyl carbon number (x-axis, range 40–60). Data points are color-coded as in panel e. A linear regression line is overlaid (Pearson r = 0.54, p = 1.26 × 10^−35^).

**Figure 4:**
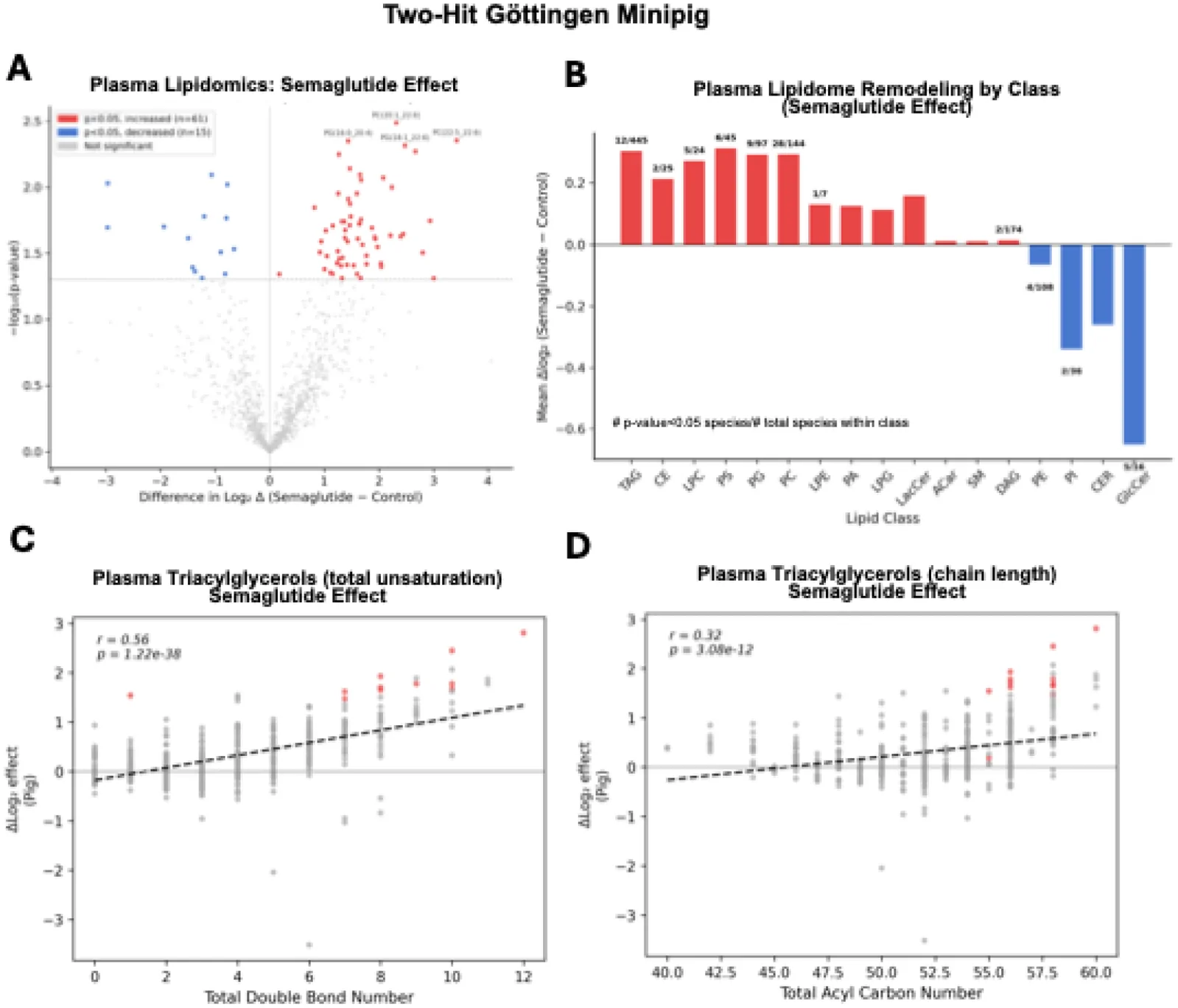
Semaglutide remodels the plasma lipidome in the porcine model of HFpEF and coronary artery disease. **a**, Volcano plot of the treatment effect on pig plasma lipid species. The x-axis shows the difference in within-animal log_2_ post-treatment change between semaglutide and control groups (Δlog_2_; semaglutide – control) and the y-axis shows −log_10_(p-value). Data points are color-coded: red dots, p < 0.05 and increased in semaglutide (n = 61 species); blue dots, p < 0.05 and decreased (n = 15 species); grey dots, not significant. **b**, Bar chart of mean Δlog_2_ treatment effect (semaglutide – control) for each of the 17 measured lipid classes in pigs. Red bars indicate a net positive treatment effect; blue bars indicate a net negative treatment effect. Numbers above each bar denote the count of nominally significant species (p < 0.05) relative to the total number of species measured in that class. Lipid classes displayed are the same 17 classes as in Figure 3b. **c**, Scatter plot of the Δlog_2_ treatment effect for individual TAG species (y-axis) versus total acyl chain double bond number (x-axis, range 0–12). A linear regression line is overlaid (Pearson r = 0.56, p = 1.22 × 10^−38^). **d**, Scatter plot of the Δlog_2_ treatment effect for individual TAG species (y-axis) versus total acyl carbon number (x-axis, range 40–60). A linear regression line is overlaid (Pearson r = 0.32, p = 3.08 × 10^−12^). n = 5 per group.

## Data Availability

The data supporting the findings of this study are available from the corresponding author upon reasonable request.
